# Biomechanical assessment of the stability of osteochondral grafts implanted in porcine and bovine femoral condyles

**DOI:** 10.1177/0954411919891673

**Published:** 2019-12-04

**Authors:** Philippa Bowland, Raelene M Cowie, Eileen Ingham, John Fisher, Louise M Jennings

**Affiliations:** Institute for Medical and Biological Engineering, University of Leeds, Leeds, UK

**Keywords:** Osteochondral graft, knee, mosaicplasty, biomechanics, primary stability, press-fit

## Abstract

Osteochondral grafts are used clinically to repair cartilage and bone defects and to restore the congruent articulating surfaces of the knee joint following cartilage damage or injury. The clinical success of such osteochondral grafts is heavily reliant on the biomechanical and tribological properties of the surgical repair; however, a limited number of studies have investigated these factors. The aim of this study was to evaluate the influence of graft harvesting and implantation technique as well as bone properties on the primary stability of press-fit implanted osteochondral grafts using a series of uniaxial experimental push-in and push-out tests. Animal (porcine and bovine) knees were used to deliver models of different bone properties (elastic modulus and yield stress). The study showed the graft harvesting method using either a chisel or drill-aided trephine to have no influence on primary graft stability; however, the preparation technique for the graft recipient site was shown to influence the force required to push the graft into the host tissue. For example, when the length of the graft was equal to the recipient site (bottomed), the graft was more stable and dilation of the recipient site was shown to reduce short-term graft stability especially in immature or less dense bone tissue. The push-out tests which compared tissue of different skeletal maturities demonstrated that the maturity of both the graft and host bone tissue to influence the stability of the graft. A higher force was required to push out more skeletally mature grafts from mature bone tissue. The study demonstrates the importance of surgical technique and bone quality/properties on the primary stability and ultimately, the success of osteochondral grafts in the knee.

## Introduction

Osteoarthritis affects more than 8 million people in the United Kingdom with the knee being the most commonly afflicted joint.^1,2^ Conservative treatments such as pharmacological interventions, physiotherapy and weight loss are frequently used to ease symptoms.^3^ However, when these approaches are not efficacious, surgical interventions may be appropriate. For patients with isolated chondral lesions with no additional comorbidities of the joint, there are a number treatments which may be adopted that aim to restore the congruent articulating surfaces. These include microfracture which stimulates fibrous tissue repair, transplantation of either autologous or allogeneic osteochondral grafts or cell-based approaches such as autologous chondrocyte implantation (ACI)/matrix-induced autologous chondrocyte implantation (MACI).^4^ Where there is extensive cartilage degeneration, total or partial joint replacement may be more appropriate.^5,6^

The presence of a focal defect in the cartilage surface may cause high contact stresses^7,8^ which often leads to tissue damage. Osteochondral grafts implanted flush to the cartilage surface can be used to reconstruct the articulating surface which can restore the contact pressure and area of the natural joint surface.^8–10^ The clinical application of osteochondral grafts in the knee involves implantation of either single or multiple (mosaicplasty) autologous or allogeneic grafts into osteochondral defects to restore the articular surface.^5^ The advantage of osteochondral grafting over microfracture or cell-based therapies is the immediate restoration of the articulating surfaces of the joint.^4^ However, a number of factors limit the clinical use of osteochondral grafts. These include tissue availability, donor site morbidity, disparity in congruency between the graft and host and lack of integration between the graft and host tissue.^4^ Despite the clinical adoption of biological interventions such as osteochondral grafting especially in young patients for whom a joint replacement may not be appropriate, a limited number of studies have been carried out to investigate the mechanical stability and tribological performance of these grafts.^11,12^ The success of osteochondral grafting in restoring the biomechanics and biotribology of the joint has been shown to depend on the restoration of the articulating surfaces.^11^ This study focuses on how variations in surgical technique and bone properties influence the primary stability of the graft prior to integration between the graft and host tissue.

**Figure 1.**
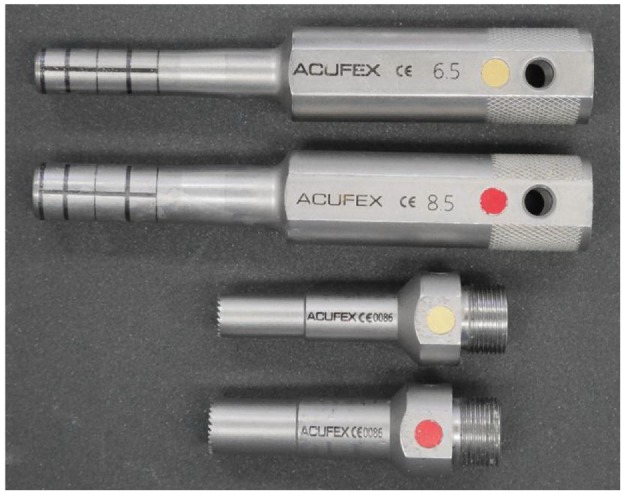
Top: tubular chisel; bottom: drill-aided trephine used to take 6.5- and 8.5-mm-diameter osteochondral plugs using the Smith and Nephew Acufex™ Mosaicplasty surgical kit.

The surgical techniques considered were the method for harvesting the graft and the preparation of the defect site. There are two commonly used methods for harvesting graft tissue either using a tubular chisel or a drill-aided trephine (Figure 1). The trephine has a serrated cutting edge and for the surgical kit used (Acufex™ Mosaicplasty surgical kit; Smith and Nephew, MA, USA) is an optional method for taking grafts from hard tissue.^13^ The more commonly used chisel method involves driving the tubular chisel into the tissue perpendicular to the cartilage surface using a hammer to a depth of ∼15 mm. The corer is then toggled to break the osteochondral plug which can then be removed and ejected from the corer.^14–16^ When preparing the recipient site, first, the recipient site is drilled, then for the surgical kit used, it is a standard practice to use a conical dilator to compact the surrounding subchondral bone, and slightly widen the recipient hole at the articular surface.^15–19^ The influence of dilation of the recipient site was investigated as well as the depth of the recipient site in relation to the length of the graft. This investigation was carried out using grafts implanted in the femur of porcine and bovine tissues as opposed to human tissue. The use of animal tissue has advantages including, greater consistency between tissue samples which reduces biological variability, availability of tissue and different animal species of varying skeletal maturities give models of varying bone properties such as yield stress and elastic modulus which allows different bone characteristics to be investigated.^20,21^ The porcine tissue, from relatively young animals where the bone mineral density (BMD) is still increasing^22,23^ represented bone with an elastic modulus and yield stress at the lower limit of that of patients who would potentially undergo osteochondral grafting as a treatment for focal osteochondral lesions; the bovine tissue with a higher elastic modulus and ultimate strength, potentially exceeding that of human bone^24–26^ represented tissue from more skeletally mature patients with a higher BMD.

The aim of the study was to assess the mechanical stability of osteochondral grafts using a series of experimental uniaxial push-in and push-out tests investigating different parameters associated with the surgical technique used for graft harvesting and implantation, and bone properties. The study aimed to address the following research questions: (1) Does the method of harvesting the graft either using a drill-aided trephine or a chisel influence the initial graft stability? (2) Does the preparation of the recipient site for the graft influence the initial graft stability? in terms of (a) the depth of the recipient site in relation to the graft length and (b) the use of dilation of the recipient site? (3) How do bone properties of the graft and/or host tissue influence graft stability?

## Methods

Skeletally mature bovine femurs from 18- to 24-month animals and 4- to 6-month skeletally immature porcine femurs with intact femoral condyles were used in this study to deliver models of different bone properties such as yield stress and elastic modulus.^20,21^ All tissues were sourced from a local abattoir supplying the food trade. Tissue samples were kept hydrated throughout the preparation procedures using phosphate buffered saline (PBS; MP Biomedicals LLC, UK) and stored until required for testing in PBS-soaked tissue paper at −20 °C. Samples were removed from storage prior to testing and thawed at room temperature.

### Push-in test

#### Sample preparation

The aim of the push-in test was to determine the force required to push a single osteochondral graft below congruency. Femurs (bovine or porcine) were cemented into a jig which allowed the inclination angle of the bone to be adjusted. Grafts of 6.5 mm (bovine or porcine) or 8.5 mm (bovine) in diameter and 10 mm in length were taken from the weight-bearing regions of the femoral condyles using the Acufex™ Mosaicplasty surgical kit using either a hammer and chisel or drilled using a trephine according to the manufacturer’s guidelines. Grafts were implanted into the weight-bearing regions of the medial femoral condyles. The recipient hole was prepared to a known depth using the drill, dilator and tamp from the Acufex™ Mosaicplasty surgical kit. The graft was then inserted into the recipient site flush with surrounding cartilage. Four grafts were positioned in each bovine femur and three in each porcine femoral condyle. Throughout the tests, the surface of the cartilage was kept hydrated with PBS.

#### Mechanical testing

**Figure 2.**
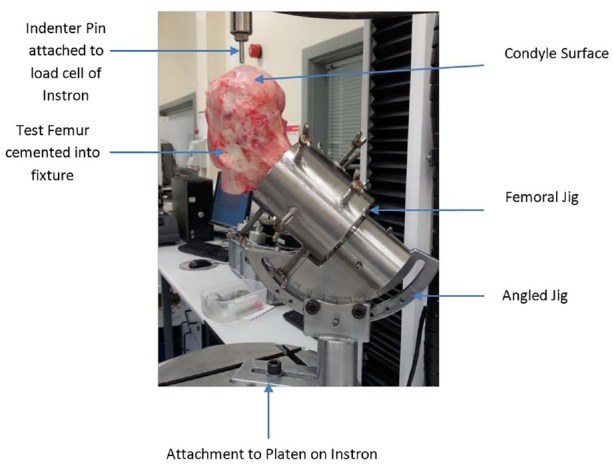
Experimental setup for push-in tests using a bovine femur.

The test method used was adapted from previous work by Kock et al.^27^ and Lowery,^28^ as shown in Figure 2. An Instron 3365 (Instron, MA, USA) materials testing machine fitted with a 5-kN load cell and a large stainless steel base platen was used. A conical 5-mm-diameter indenter was attached to the movable cross-head of the Instron materials testing machine, and the inclination angle of the femur with implanted grafts adjusted so that the surface of the graft was perpendicular to the indenter. Grafts were compressed at 1 mm/min until 4 mm below congruency, the output force and displacement were recorded at a rate of 10 samples/s. All tests were carried out at room temperature.

The experimental groups are shown in Table 1. A minimum of *n* = 5 was carried out for each experimental group and the uneven sample sizes were due to failed samples. A test was considered to have failed if one of the following occurred: 4 mm below congruency was not achieved, if the indenter did not remain perpendicular to the surface of the graft or if the indenter contacted the side walls of the recipient hole. For each test, the diameter of the graft used, the depth of the recipient site and the type of tissue were based on the research question to be answered.

**Table 1.** Experimental groups for push-in tests relating to the influence of graft harvesting (research question 1) and the preparation of the recipient site (research question 2).

Researchquestion	Tissue used for graftand recipient site	Osteochondral graft			Recipient site		Samplenumber
		Length (mm),bottomed/unbottomed	Diameter(mm)	Method of harvest	Depth(mm)	Dilated?	
1	Bovine	10, bottomed	6.5	Chisel	10	Yes	7
	Bovine	10, bottomed	6.5	Trephine	10	Yes	11
2a	Bovine	10, bottomed	8.5	Chisel	10	Yes	12
	Bovine	10, unbottomed	8.5	Chisel	15	Yes	10
2b	Porcine	10, unbottomed	6.5	Chisel	15	Yes	5
	Porcine	10, unbottomed	6.5	Chisel	15	No	6

### Push-out test

**Figure 3.**
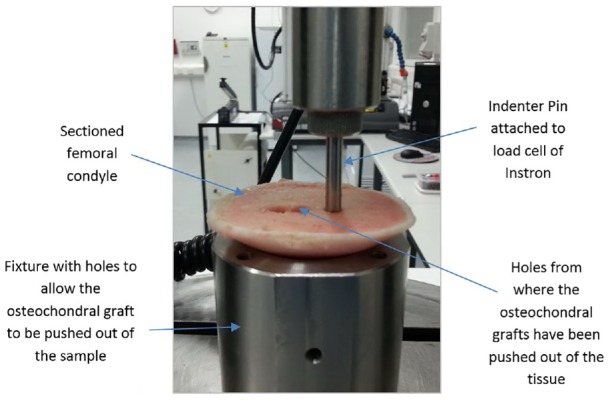
Experimental setup for push-out tests.

The aim of the push-out test was to determine the force required to displace an osteochondral graft from within the host tissue immediately after implantation. Grafts were inserted into recipient holes positioned flush to the cartilage surface in the weight-bearing region of the medial femoral condyle, as described for the push-in tests. The distal part of the condyle was then cut away from the rest of the femur at the level of the base of the grafts. This allowed the base of the grafts to be accessed. The section of bone with the grafts was placed cartilage-side down on a hollow support fixture which allowed the graft to be pushed out of the host bone tissue without interference from the fixture. A 5-mm indenter was attached to the Instron materials testing machine which was used to push the graft from its base out of the back of the tissue using a rate of 1 mm/min (Figure 3). The maximum push-out force (*F*_max_) was measured. The experimental groups are described in Table 2, the uneven sample numbers are due to failed tests which occurred as a result of the indenter contacting the edge of the recipient site.

**Table 2.** Experimental groups for push-out tests relating to the influence of bone properties on graft stability (research question 3).

Osteochondral graft				Recipient site	Sample number
Tissue type	Length (mm)	Diameter (mm)	Method of harvest	Tissue type	
Bovine	10	6.5	Chisel	Bovine	11
Porcine	10	6.5	Chisel	Bovine	7
Porcine	10	6.5	Chisel	Porcine	5

For the push-in tests, the mean load ± 95% confidence limits was calculated for each experimental group at 0.25 mm displacement intervals. For the push-out tests, the maximum mean push-out force ± 95% confidence limits was calculated. Statistical analysis was carried out using one-way analysis of variance (ANOVA) with a post hoc Tukey’s test in SPSS. The experimental groups investigated for each research question were compared. For clarity, in the push-in tests, this was carried out at 0.25 mm displacement intervals. Significance was taken at *p* < 0.05.

The data associated with this article are openly available from the University of Leeds data repository.^29^

## Results

### Influence of graft harvesting method

**Figure 4.**
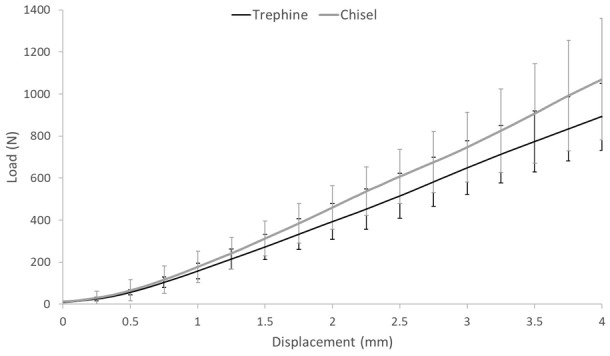
Compressive load measured against displacement for 6.5-mm-diameter bottomed osteochondral allografts harvested with either a drill-aided trephine (*n* = 11) or a chisel (*n* = 7). Tests were conducted using an all-bovine model. Data plotted as mean ± 95% confidence limits.

Harvesting grafts using a drill-aided trephine was more technically difficult than using a chisel and there was greater potential to damage the cartilage surface on both the graft and the surrounding recipient site. In terms of graft stability, there was no significant difference (*p* > 0.05) at any level of displacement below congruency in the force required to displace bottomed 6.5-mm-diameter grafts harvested from bovine tissue using either a chisel or a trephine (Figure 4).

### Preparation of recipient site

To investigate whether the preparation of the defect site influenced graft stability, first, the depth of the recipient site in relation to the length of the graft was investigated and second the influence of dilation of the recipient site on the primary graft stability was assessed.

#### Depth of recipient site

**Figure 5.**
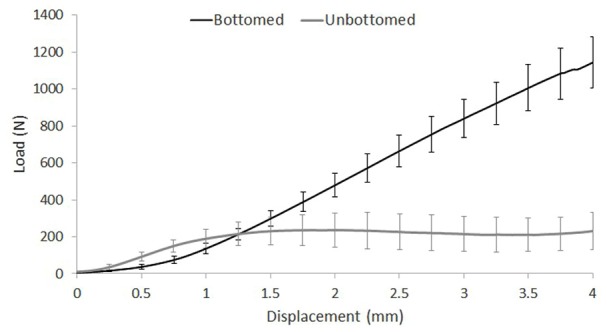
Compressive load measured against displacement for 8.5-mm-diameter bottomed (*n* = 12) and unbottomed (*n* = 10) osteochondral allografts. Tests were conducted using an all-bovine model. Data plotted as mean ± 95% confidence limits.

The load–displacement curves for bottomed grafts, those for which the length of the graft was equal to the depth of the recipient site and unbottomed grafts, for which the recipient site was deeper than the length of the graft, were markedly different (Figure 5). For unbottomed grafts, an initial increase in load was observed as the graft–host interference forces were overcome, then as the graft moved below congruency with the articulating surface, the load required to displace the graft stabilised. The test was stopped at 4 mm displacement so the graft did not reach the bottom of the recipient site. The mean force required to displace bottomed grafts was significantly higher (*p* < 0.05) compared with unbottomed grafts at all levels of displacement greater than 1.5 mm. For the bottomed grafts, from 1 to 4 mm below congruency, there was a linear rate of increase in the force required to displace the grafts.

#### Dilation of the recipient site

**Figure 6.**
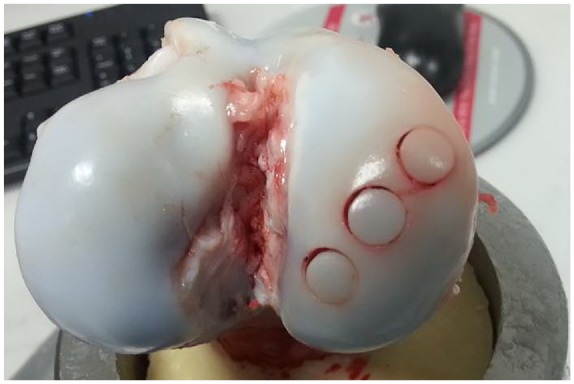
Grafts of 6.5-mm-diameter implanted into the medial femoral condyle of a porcine femur, the recipient site was dilated prior to graft implantation. The poor fit between the graft and host tissue can clearly be seen.

**Figure 7.**
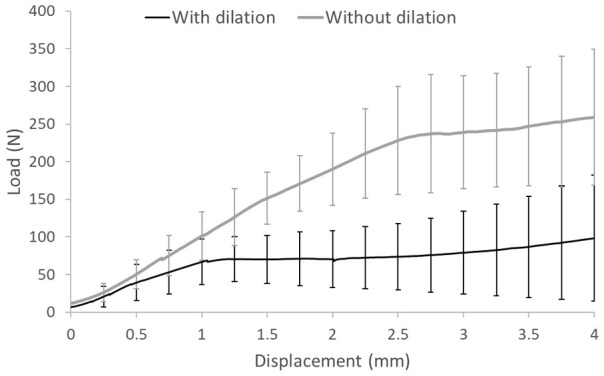
Compressive load measured against displacement for 6.5-mm-diameter osteochondral allografts either with dilation of the recipient site (*n* = 5) or without dilation of the recipient site (*n* = 6). Tests were conducted using an all-porcine model. Data plotted as mean ± 95% confidence limits.

For porcine tissue, when the recipient site was dilated, there was a poor fit between the graft and the recipient site resulting in a fissure between the edge of the recipient site and the graft (Figure 6). Without dilation of the recipient site, this fissure was not observed, there was a continuous surface over the graft and host tissue and a significantly higher push-in force was required to displace the porcine grafts at all displacements greater than 1.25 mm below congruency compared with grafts inserted with dilation. For grafts inserted with dilation, movement of the graft occurred at a lower force compared with grafts inserted without dilation (Figure 7).

### Influence of bone properties

**Figure 8.**
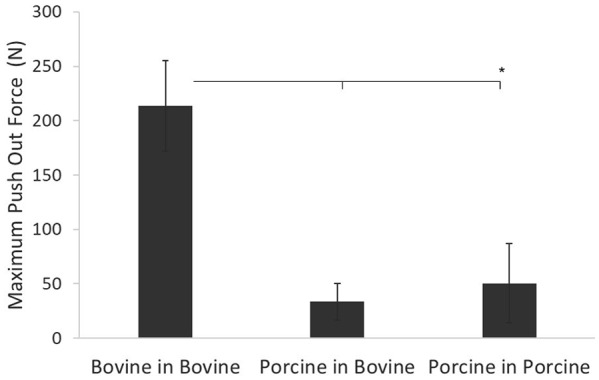
Maximum push-out force required to overcome the graft–host interface shear forces. Data plotted as mean ± 95% confidence intervals. *Denotes a significant difference (*p* < 0.05) in the group mean values when compared with the bovine in bovine group.

The mean push-out forces were 213.4 ± 41.5 N, 33.4 ± 16.8 N and 50.4 ± 36.2 N for bovine allografts, porcine xenografts (implanted in bovine tissue) and porcine allografts, respectively (Figure 8). The maximum push-out force for bovine allografts was significantly higher (*p* < 0.05) than for either the xenograft or porcine allograft groups. There was no significant difference (*p* > 0.05) between the xenograft and porcine allograft groups (Figure 8).

## Discussion

One of the key factors in determining the success of osteochondral graft surgery is the restoration of the congruent articular surface of the cartilage. The aim of this study was to investigate the primary stability of the graft immediately post surgery, prior to tissue integration. Grafts which protrude above or subside below congruency level following implantation, may induce inferior biomechanical and tribological^11,12^ conditions in the joint, potentially resulting in the onset of degenerative changes or the formation of tissue which is biomechanically and histologically inferior to native articular cartilage.^9,30,31^ This study investigated whether surgical technique or bone properties, would influence the force required to displace the graft in the host tissue.

The first variable to be considered was the surgical technique for harvesting grafts. The surgical kit used (Acufex™) gives two methods for extracting grafts, either hammering a tubular chisel into the tissue or using a drill-aided trephine. The trephine method is optional for hard tissue. The primary stability of osteochondral grafts harvested using the different methods has not previously been investigated, and this study showed there was no significant difference in the push-in forces required to displace grafts harvested using either a corer or trephine. However, using the drill method, in some samples, the cartilage either on the graft surface or surrounding the donor site was visibly damaged. Evans et al.^32^ previously reported similar findings whereby using a chisel method was shown to cause less cartilage damage than drilling the graft and Vizesi et al.^33^ showed superior healing response of defects created using a punch rather than a drill. Robert^16^ in his description of surgical technique with the single-use OATS^®^ instrumentation from Arthrex suggests the donor graft should be extracted using a mallet, never a drill. The design of the cutting edge of the chisel has also been shown to influence tissue damage and cell viability in neighbouring tissue.^34^ These studies combined suggest that while the harvesting method may not influence the stability of osteochondral grafts in situ, the potential for the method used to damage tissue and influence the long-term healing and success of the graft should be considered.

The surgical technique for graft implantation was also investigated. First, the influence of graft length compared with the depth of the recipient hole was considered. The load–displacement curves for unbottomed grafts where the recipient site was deeper than the graft length and bottomed grafts where the graft length was equal to the recipient site were noticeably different. For unbottomed grafts, the load–displacement curves showed an initial increase in load per unit of displacement over the first 1.5 mm of motion when, to initiate graft movement, the graft–host interference force must be overcome. Once the graft began to move, the force required for the graft to continue descending into the recipient site was constant resulting in a plateau in the load–displacement curve. The bottomed grafts were supported by the underlying trabecular bone, therefore, when subjected to a push-in test, they underwent confined compression resulting in a much steeper and prolonged increase in load per unit of displacement compared with the unbottomed grafts. The higher push-in force of bottomed grafts compared with unbottomed grafts is consistent with previously published research.^27,35,36^ The force required to displace the graft 4 mm below congruency was approximately three times higher in bottomed grafts compared with unbottomed grafts; this difference in magnitude force is similar to that measured by Kordás et al.^35^ in a cadaveric knee study. These results show the importance of the graft length being equal to that of the recipient site for primary graft stability.

The preparation of the recipient site was also investigated in terms of the use of dilation of the host site. With the Acufex™ Mosaicplasty surgical toolkit, dilation of the recipient site is standard procedure; however, the influence of recipient site dilation on the primary stability of osteochondral grafts implanted in tissue with different bone properties has not previously been considered. For this study, porcine tissue was used from younger, less skeletally mature animals compared with the bovine tissue used to answer the other research questions. During sample preparation, it became apparent that dilation resulted in recipient holes which appeared larger than the 6.5-mm-diameter graft and in many samples this led to a poor fit between the graft and host tissue. Grafts inserted without the use of dilation required a significantly higher (*p* < 0.001) push-in force to overcome the static friction between the graft and host than when dilation was used. When measured, the diameter of the drill bit was 6.35 mm and the dilator 6.80 mm showing the extent to which dilation enlarged the recipient site. Kordás et al.^37^ investigated the influence of dilation and showed greater graft stability with a shorter dilation length. In the porcine model, a shorter dilation length would have still resulted in a mismatch at the articulating surface, dilation was used on all bovine tissue samples and the discrepancy between the graft edge and the recipient site was not seen in this more mature tissue.

When implanting osteochondral grafts, it is important to consider how the implantation technique affects not only the primary stability of the graft but also the regenerative capacity of the graft and host tissue. High impaction forces generated either using a graft which is longer or of larger diameter than the recipient site^27,35,36^ potentially improve graft stability; however, high forces applied to the cartilage surface have also been associated with chondrocyte death and ultimately the success of the graft.^38–41^ Therefore, to achieve long-term survival of the graft, a compromise must be achieved between using a sufficiently high insertion force to achieve a good interference fit between the graft and host tissues while minimising tissue damage. This implies that clinically, the quality and properties of the tissue should therefore be considered when determining whether dilation of the recipient site is appropriate. In higher modulus bone, dilation may allow a graft to be implanted with a lower force whilst maintaining both cell viability and the congruent articulating surfaces; in lower modulus bone, there is the potential for dilation to reduce the interference fit between the graft and recipient site giving a less congruent articulating surface.

In the push-out study, the maximum forces measured for porcine grafts in porcine recipient sites (50 ± 36 N) were comparable with pull-out forces measured in previous studies by Duchow et al.^42^ (41 ± 21 N) for grafts of similar geometry. The push-out forces were lower when porcine tissue was used for either the graft or recipient site compared with a bovine-in-bovine model. The increased force required to push out the bovine grafts was attributed to the skeletal maturity of the samples, likely higher bone mineral density and higher modulus of the more mature bovine bone compared with the more skeletally immature bone and lower modulus of the porcine samples.^20–22^ These results highlight the importance of the maturity and quality of the underlying bone to achieve initial stability of the graft.

There were a number of limitations associated with this study, first, only a single graft was used, often in larger defects, multiple grafts are introduced (mosaicplasty). Single grafts have been shown to be more stable than multiple grafts due to the larger contact area between the graft and host tissue;^37^ however, the use of multiple grafts may better restore the geometry of the articulating surfaces. The test method applied a constant displacement rate which did not replicate the complicated loading and motion profiles experienced by the joint in vivo. For this study, healthy animal tissue was selected rather than human tissue to control bone properties, reduce biological variability and for tissue availability; however, this approach gave no consideration to potential disease or degeneration of the cartilage or underlying bone. Using animal tissue from different sources allowed the functional stability of osteochondral grafts in tissue of different moduli, to start to be investigated. The porcine tissue from relatively young, skeletally immature animals^22,23^ represented bone with an elastic modulus and yield stress at the lower limit of that of patients who may undergo osteochondral grafting as treatment for focal osteochondral lesions; the skeletally mature bovine tissue with a higher elastic modulus and ultimate strength, potentially exceeding that of human bone^24–26^ represented tissue patients with a higher BMD. This study therefore shows that the quality and properties of the host and recipient tissue influence the clinical outcome; however, future work is necessary to determine the applicability of the animal models to human tissue. Furthermore, this study shows the importance of considering the properties of any synthetic or decellularised osteochondral scaffold combined with the properties of the host tissue on the resulting stability. Finally, the study considered only the initial stability of the grafts following implantation, within the first approximately 4 weeks following implantation, the stability of the graft is reliant on the press-fit mechanism,^19^ after this time period, bone remodelling starts to give the graft additional stability. Therefore, although longer term integration of the graft and host tissue has not been considered in this study, both the primary press-fit stability and the longer term integration of bone tissue influence the success of the treatment.

## Conclusion

Understanding factors which influence the initial stability of osteochondral grafts is important for the longer term restoration of the congruent articulating surfaces and the success of the osteochondral graft. This study highlights the importance of surgical technique on the initial biomechanical stability of osteochondral grafts. The push-in forces were higher suggesting that the graft would be more stable when the length of the graft was equal to the depth of the recipient hole (bottomed grafts) and bone tissue was more mature. In terms of graft stability, the method for harvesting the graft did not influence the push-in force; however, dilation of the host tissue was shown to influence the interference fit between graft and host in lower modulus bone.
